# A household survey of medicine storage practices in Gondar town, northwestern Ethiopia

**DOI:** 10.1186/s12889-017-4152-8

**Published:** 2017-03-09

**Authors:** Fitsum Sebsibe Teni, Abdrrahman Shemsu Surur, Assefa Belay, Dawit Wondimsigegn, Dessalegn Asmelashe Gelayee, Zewdneh Shewamene, Befikadu Legesse, Eshetie Melese Birru

**Affiliations:** 1 Department of Pharmaceutics and Social Pharmacy, School of Pharmacy, College of Health Sciences, Addis Ababa University, Addis Ababa, Ethiopia; 2 Department of Pharmaceutical Chemistry, School of Pharmacy, College of Medicine and Health Sciences, University of Gondar, Gondar, Ethiopia; 3 Department of Pharmacology, School of Pharmacy, College of Medicine and Health Sciences, University of Gondar, Gondar, Ethiopia; 4 Department of Pharmaceutics and Social Pharmacy, School of Pharmacy, College of Medicine and Health Sciences, University of Gondar, Gondar, Ethiopia; 5 Department of Public Health Sciences, Karolinska Institute, Stockholm, Sweden

## Abstract

**Background:**

Household surveys are crucial to get accurate information on how medicines are acquired, and used by consumers, as they provide the best evidence in the area. The objective of this study was to document household medicine storage practices in Gondar town, northwestern Ethiopia.

**Methods:**

A cross-sectional household survey was conducted from April 5 to May 6, 2015. In the study, 809 households were surveyed from four sub-cities in the town selected through multistage sampling with 771 included in the final analysis. Data on the extent of storage, storage conditions, sources of medicines and their current status among others were collected through structured interviews and observations. The data were entered in to Epidata version 3.1, exported to and analyzed using Statistical Packages for Social Sciences (SPSS) version 21.

**Results:**

Of the 771 households in the study, 44.2% stored medicines. Presence of family members with chronic illness(es) and higher levels of household incomes predicted higher likelihood of medicine storage. In the households which allowed observation of stored medicines (*n* = 299), a mean of 1.85 [SD = 1.09] medicines per household were found. By category, anti-infectives for systemic use (23.9%), medicines for alimentary tract and metabolism (19.2%) and those for cardiovascular system (17.7%) ranked top. Among individual medicines stored, diclofenac (10.7%), paracetamol (9.9%) and amoxicillin (8.0%) were on top of the list. Dispensaries (97.8%) and physicians (83.5%) were almost exclusive sources of medicines and advices/orders for medicines respectively. Nearly two-thirds of the medicines found were on use and a vast majority (76.5%) were stored in chests of drawers. Proportion of expired medicines was very low (3.14%).

**Conclusions:**

The use of physicians’ and pharmacists’ advice to get medicines; use of dispensaries as principal sources, large proportion of medicines being in use and very low proportion of expiry showed good practices. However, storage places of medicines were not purpose built. Encouraging good practices through continued medicine use education and advocating appropriate medicine storage in medicine cabinets is required to improve storage conditions and consequent use of medicines.

**Electronic supplementary material:**

The online version of this article (doi:10.1186/s12889-017-4152-8) contains supplementary material, which is available to authorized users.

## Background

Medicines found in households are commonly sourced from health institution dispensaries through prescriptions or from pharmacies with or without prescriptions. These medicines could be in use for current illnesses or remaining from past use [1]. Expenditure on medicines takes up from nearly a third to two-fifth of health care spending in developing countries. Purchases made by individual consumers constitute a major proportion of the spending, mostly for self-medication and rarely on prescriptions. Getting a good understanding of the individual consumer choices and decisions is crucial for intervention in ensuring efficient resource use. This is important as out of pocket spending by individuals is an important component of expenditures on medicine [2, 3].

While indicators of access to medicines are most commonly taken from health facilities and medicine retail outlets, there is little information from consumers. Although data obtained through indicators measured at health institution levels are vital, household surveys are crucial to get accurate information on how medicines are acquired and used by consumers. Such studies are important as they are the best sources of evidence in the area [1, 4].

The extent, source and storage conditions of medicines kept at household level provide crucial information on access and medicine use. Globally studies on household use and storage of medicines have been conducted in different countries. In many of the studies significant proportions of households stored medicines in some cases with all of them keeping medicines [5–18]. In the same and other studies, considerable levels of unused and expired medicines kept at households were reported [19–25]. These medicines were stored at home reportedly due to patient deaths, recovery from disease, expiry as well as changes in medicine [26].

In Ethiopia, so far, few studies were conducted at household level to document use and storage practice of medicines. The findings showed the proportion of households which stored medicines ranged from one-fifth to more than one-half in different parts of the country [27–29]. Apart from these, medicine storage practices at household level remain unstudied. Evidence on extent and condition of storage of medicines is crucial to inform actions toward ensuring rational use. So, the objective of this study was to document household medicine storage practices among households in Gondar town in northwestern Ethiopia. It involved comprehensive assessment of the extent of storage, source, types, duration, use status as well as expiry status of the stored medicines.

## Methods

### Study area and design

In this study, a household survey on medicine storage practice among residents of Gondar Town in northwestern Ethiopia, was conducted. The town is located about 750 km away from the capital Addis Ababa, and was home to 224,000 population in 2014/15 according to Central Statistical Agency of Ethiopia (CSA) [30]. The administrative division of the town includes a total of 24 ‘kebeles’ (the smallest administrative division), among which 12 in the urban areas are classified as sub-cities. The other 11 ‘kebeles’ and one special ‘kebele’ make up the rural areas of the town. The present study was undertaken in the urban areas of the town (Gondar Town Administration. Administrative classification of kebeles in Gondar town. 2014, unpublished).

The town has various public and privately owned health institutions. The public ones include a specialized referral university hospital and a number of health centers. There are also nearly 50 clinics and one hospital in the private sector. More than 50 medicine retail outlets are also found concentrated in the urban areas of the town (Gondar Town Health Bureau. Report on the number of medicines retail outlets in Gondar town 2014, unpublished), (Amhara Regional Health Bureau. Health Management Information System (HMIS) Implementation at private facilities: advocacy 2013, unpublished).

### Sampling

The number of households included in the study was determined using a single population proportion formula; assuming the proportion (*p*) of households with at least one medicine at the time of data collection to be 50%, for maximum possible sample. The margin of error (δ) was taken to be 5% and the *z*_1 − ∝_ at 95% confidence interval (CI) was set at 1.96. Based on this the sample size was calculated using the formula: $\left[ N=\frac{{\left({z}_{1-\propto}\right)}^2 \times p\left(1- p\right)}{\delta^2}\right]$ [31]. After taking in to account a contingency of 5% and a design effect of 2, the final sample size was calculated to be 809 households.

A multistage sampling procedure was followed in sampling the households. In the first stage four sub-cities which accounted for one-third of the sub-cities in the town were selected by simple random sampling. Then the calculated number of households was equally divided into the four kebeles. In the second stage, from the selected sub-cities households were sampled by random selection from the list of households.

### Data collection instrument, process and management

An instrument composed of a structured interviewer-administered questionnaire and a structured observation checklist was used for data collection. It was developed by adapting tools used in previous studies and guidelines [2, 12]. The adaptation involved including parts/questions of the instruments which were relevant to answer the objectives of this study. It was first prepared in English and translated in to Amharic, a language spoken in the study area, and then back translated into English to make sure it retained the intended meaning. The data collection tool is provided as a supplementary file to this manuscript [Additional file 1].

The instrument contained questions on the socio-demographic profile of the respondents and their households, the overall health situation in the household, and specifics of medicines stored. These included current use status of the medicine(s), illnesses the medicine(s) were acquired for, prescription status and source. The structured obsevation focused on the name, packaging, storage conditions and others. The instrument was pretested on 50 respondents prior to the actual data collection, which were excluded from the final analysis, and pertinent modifications were instituted based on the finding.

Data were collected from April 5 to May 6, 2015 by four data collectors, with a diploma level of qualification in pharmacy, after a thorough one day training. The training focused on the data collection instruments and the appropriate approaches required in interacting with and securing consent of respondents.

Respondents approached in the study were adults available in the selected households during data collection. Whenever more than one willing adults were found, priority was given to the one deemed more informed on the health related issues of the household. In case of unavailability of eligible respondents a second visit was made. If this failed, the household next to it was included in the survey instead.

### Data entry, analysis and interpretation

The collected data was coded and entered using Epidata version 3.1 (Epidata Association, Odense, Denmark). It was then exported to and analyzed by using Statistical Packages for Social Sciences (SPSS) version 21 (IBM Corp. IBM SPSS Statistics for Windows, Armonk, NY: IBM Corp. Released 2012). Data on medicines recorded from the observation in the households were categorized using the World Health Organization (WHO) Anatomical Therapeutic Chemical (ATC) classification system level one [32]. Descriptive analyses were performed by frequency and mean, with results presented in tables and bar charts. In the analysis of the association between socio-demographic as well as other related variables and medication storage practices, independent samples T tests, one way analysis of variance (one way ANOVA) and binary logistic regression tests were undertaken. A *p*-value cut off point of 0.05 at 95% CI was used to determine statistical significance of association.

## Results

### Socio-demographic profile of respondents and their households

From the 809 households in the study, surveys of the 771 were deemed complete and included in the final analysis making the response rate 95.3%. Of the participants of the study who represented their respective households, upwards of three quarters (76.3%) and two-fifths (40.9%) were female and those in the age group of 18 to 29 years, respectively. Nearly three-fourths (73.3%) followed Orthodox Christianity and almost all (90.3) were Amhara in their ethnic identity (Table 1).

**Table 1:** Socio-demographic characteristics of respondents and their households, Gondar Town, 2015

Variable		Frequency (%)
Sex	Male	183 (23.7)
	Female	588 (76.3)
Age (years)	18–29	315 (40.9)
	30–39	177 (23.0)
	40–49	127 (16.5)
	50–59	68 (8.8)
	60+	84 (10.9)
Religion	Orthodox Christianity	565 (73.3)
	Islam	159 (20.6)
	Protestantism	31 (4.0)
	Others^a^	16 (2.1)
Ethnicity	Amhara	696 (90.3)
	Tigre	47 (6.1)
	Others^b^	28 (3.6)
Educational status	Can’t read or write	162 (21.0)
	Can read and write	87 (11.3)
	Primary education	98 (12.7)
	Secondary education	249 (32.3)
	College/university education	175 (22.7)
Highest education level in family	Reading and writing	26 (3.4)
	Primary education	94 (12.2)
	Secondary education	234 (30.4)
	College/university education	417 (54.1)
Occupation	Not working/unemployed	36 (4.7)
	Housewife	311 (40.3)
	Student	49 (6.4)
	Retiree	30 (3.9)
	Government employee	106 (13.7)
	Private company employee	95 (12.3)
	Merchant	141 (18.3)
	Farmer	3 (0.4)
Family’s monthly income (USD)	Up to 50	164 (21.3)
	51 to 100	137 (17.8)
	101 to 150	110 (14.3)
	151 to 200	76 (9.9)
	200 to 250	80 (10.4)
	>250	56 (7.3)
	Not disclosed	148 (19.2)

^a^ Judaism, Catholicism

^b^ Qimant, Oromo

Nearly a third (32.3%) and more than a fifth (22.7%) of the respondents were at secondary and college/university education level, respectively. In the overwhelming majority (84.5%) of the households, the highest level of education reported was secondary or higher education level. Housewives accounted for the largest proportion (40.3%) of respondents as to occupational status. In terms of income, more than one-fifth (21.3%) of the households reported monthly earnings of 50 United States Dollars (USD) followed by those earning 51 to 100 USD (17.8%). Nearly a fifth of the households (19.2%) did not disclose their monthly earnings (Table 1).

### Number of medicines stored in the households

**Fig. 1:**
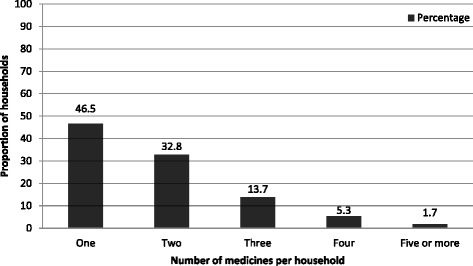
Percentage distribution of households by number of medicines stored (*n* = 299 households)

Of the total 771 households included in the analysis, 341 (44.2%) had kept medicines at home during data collection. However, 42 (12.3%) of these households were unable or unwilling to show the medicines. In the 299 households where it was possible to observe medicines stored, an average of 1.85 [SD = 1.09] medicines per household were found. These were constituted by 93 different medicines. Looking at the number of medicines stored, about half (46.5%) of the households stored one medicine (Fig. 1).

Based on independent samples *T* test, a statistically significant higher mean number of medicines were stored in households where family members with chronic illnesses (*p*-value < 0.01) lived. However, presence of a health professional in the household, did not show such a difference (Table 2).

**Table 2:** Statistical (independent T) tests assessing difference in the mean number of medicines stored by household situations, Gondar town, 2015 (*n* = 299)

Variable	Number of medicines stored	
	Mean (SD)	*p*-value
Member with chronic illness		
Yes	2.15 (1.294)	<.001^*^
No	1.62 (0.842)	
Presence of a health professional in the household		
Yes	1.84 (0.943)	0.929
No	1.85 (1.119)	

*p value < 0.05

Monthly income of a family and highest education levels attained in households did not show a statistically significant difference in the number of medicines kept at home (Table 3).

**Table 3:** Statistical (One-way ANOVA) tests assessing difference in the mean number of medicines stored by household situations, Gondar town, 2015 (*n* = 299)

Variable	Number of medicines stored	
	Mean (SD)	*p*-value
Family’s monthly income (USD)		
Up to 50	1.74 (0.953)	0.785
51 to 100	1.88 (1.036)	
101 to 150	1.80 (0.808)	
151 to 200	1.92 (1.025)	
200 to 250	1.76 (0.932)	
> 250	1.78 (0.751)	
Not disclosed	2.11 (1.909)	
Highest education level in the family		
Reading and writing	1.60 (1.342)	0.952
Primary education	1.79 (0.918)	
Secondary education	1.85 (0.969)	
College/university education	1.86 (1.152)	

### Types and purposes of medicines stored

**Fig. 2:**
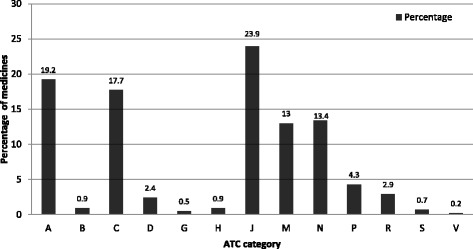
Percentage distribution of medicines at level one ATC classes stored in households, Gondar town, 2015 (*n* = 553 medicines); Legend: A = Alimentary tract and metabolism, B = Blood and blood forming organs, C = Cardiovascular system, D = Dermatologicals, G = Genitourinary system and sex hormones, H = Systemic hormonal preparations excluding sex hormones and insulins, J = Antiinfectives for systemic use, M = Musculo-skeletal system, N = Nervous system, P = Antiparasitic products, insecticides and repellents R = Respiratory system S = Sensory organs V = Various

On the basis of ATC classification, anti infectives for systemic use ranked first accounting for nearly a quarter (23.9%) of the medicines kept at home. These were followed by medicines for alimentary tract and metabolism (19.2%) and those for cardiovascular system (17.7%) (Fig. 2).

Diclofenac, paracetamol and amoxicillin accounted for the most frequently stored individual medicines with proportions of 10.7%, 9.9% and 8.0% in that order. Only eight among the 93 different medicines kept in the households made up half (50.5%) of the total 553 medicines found (Table 4).

**Table 4:** The most frequently stored medicines in households, Gondar town, 2015 (*n* = 553 medicines)

Medicine name	Frequency (%)
Diclofenac	59 (10.7)
Paracetamol	55 (9.9)
Amoxicillin	44 (8.0)
Hydrochlorothiazide	36 (6.5)
Enalapril	25 (4.5)
Metformin	23 (4.2)
Glibenclamide	22 (4.0)
Omeprazole	15 (2.7)
Insulin	13 (2.4)
Metronidazole	13 (2.4)
Nifedipine	13 (2.4)
Cotrimoxazole	12 (2.2)
Ciprofloxacin	10 (1.8)
Doxycycline	8 (1.4)
Tetracycline	8 (1.4)

The medicines observed in the households were reportedly acquired for managing different health problems/illnesses. These included 39 different illnesses/conditions reported by the households with a total frequency of 452. Of the ten most frequently reported illnesses or conditions for which medicines were taken and kept in the households, headache (16.4%), hypertension (13.9%) and diabetes mellitus (10.8%) ranked in the first three (Table 5).

**Table 5:** The ten most frequently reported illnesses/conditions for which medicines were kept in the households, Gondar Town, 2015 (*n* = 452 illnesses/conditions)

Reported illness/condition	Frequency (%)
Headache	74 (16.4)
Hypertension	63 (13.9)
Diabetes mellitus	49 (10.8)
Unspecified	29 (6.4)
Fever	26 (5.8)
Eye problem	18 (4.0)
Tonsilitis	16 (3.5)
Peptic ulcer disease	15 (3.3)
Asthma	13 (2.9)
Pain	13 (2.9)

### Storage place, current status and sources of medicines

Of the total 553 medicines stored, more than three quarters (80.8%) were of solid dosage forms. More than half (53.3%) of the medicines were on use by the persons for which they were originally acquired; while about a sixth (16.1%) of them were kept with no purpose in the households (Table 6).

**Table 6:** Features of medicines stored in households, Gondar town, 2015 (*n* = 553)

Variable		Frequency (%)
Dosage forms	Solid	447 (80.8)
	Semisolid	14 (2.5)
	Liquid	92 (16.6)
Current status of medicine	On use by the person originally intended for	295 (53.3)
	On use by another person	30 (5.4)
	Kept for future use	139 (25.1)
	Kept with no purpose	89 (16.1)
Storage place	Drawer	423 (76.5)
	Refrigerator	29 (5.2)
	Table	30 (5.4)
	Bag/purse	22 (4.0)
	Pockets on cloth	11 (2.0)
	Others^a^	38 (6.9)
Source of advice for medicine	Self-initiated	51 (9.2)
	Pharmacist	40 (7.2)
	Physician	462 (83.5)
Source of medicines	Government health institution dispensary	251 (45.4)
	Private medicine retail outlets	228 (41.2)
	Aid organization pharmacy	62 (11.2)
	Others^b^	12 (2.2)
Reason for choosing source	Short distance/proximity	240 (43.4)
	Quality of services	35 (6.3)
	Urgency to medicines	45 (8.1)
	Had follow up at source	82 (14.8)
	Availability of medicines	15 (2.7)
	Fairness of price	76 (13.7)
	Others^c^	59 (10.7)

^a^ by window, under mattress, hung in plastic bags

^b^ private clinic, sent from abroad

^c^ free of fee services, relationships, agreement with employers

The vast majority (83.5%) of the medicines observed were reported to be acquired through physician prescriptions; while nearly a tenth (9.2%) were gained through request by individuals taking the medicines or by family members. As to sources of medicines, government run health institution dispensaries (health centers and the hospital) (45.4%) and private medicine retail outlets (pharmacies and drug stores) (41.2%) were reported to be principal sources. The most commonly cited reasons for acquiring medicines from the preferred sources included distance from the source, mentioned by nearly half of the respondents (43.4%). Chest of drawers in living rooms and bed rooms were spots of storage of medicines in more than three quarters (76.5%) of the households (Table 6).

The medicines found were kept from one to as long as 730 days with a median of 15 days (Inter-quartile range = 23 days). Among the total number of medicines, 541 had their expiry dates recorded on their packaging. Of these, 17 (3.14%) were expired.

### Predictors of medicine storage in the households

Binary logistic regression tests showed that households with a family member having chronic illness were nearly 15 times (Adjusted OR (AOR) = 14.824, 95% CI = 9.072-24.222) more likely to keep medicines in their households compared to those with no such members after controlling for other variables. Higher income levels were also associated with increased likelihood of keeping medicines in the households with statistical significance in specific income categories compared to households earning up to 50 USD per month (Table 7).

**Table 7:** Binary logistic regression test for predictors of presence of medicines in households, Gondar town, 2015 (*n* = 771)

Variable	Medicines in household		OR (95% CI)	AOR (95% CI)
	Yes (%)	No (%)		
Highest level of education in the family				
Reading and writing	6 (23.1)	20 (76.9)	1	1
Primary education	22 (23.4)	72 (76.6)	1.019 [0.364-2.852]	1.120 [0.343-3.662]
Secondary education	98 (41.9)	136 (58.1)	2.402 [0.930-6.202]	2.582 [0.858-7.771]
College/university education	215 (51.6)	202 (48.4)	3.548 [1.397-9.013]^*^	2.542 [0.833-7.756]
Presence of a health professional in household				
Yes	57 (52.8)	51 (47.2)	1.492 [0.992-2.243]	0.853 [0.513-1.418]
No	284 (42.8)	379 (57.2)	1	1
Persons with chronic illness in household				
Yes	150 (86.7)	23 (13.3)	13.897 [8.674-22.266]^*^	14.824 [9.072-24.222]^*^
No	191 (31.9)	407 (68.1)	1	1
Household monthly income (USD)				
Up to 50	54 (32.9)	110 (67.1)	1	1
51–100	63 (46.0)	74 (54.0)	1.734 [1.086-2.769] ^*^	1.402 [0.814-2.414]
101–150	59 (53.6)	51 (46.4)	2.357 [1.434-3.872] ^*^	1.813 [1.002-3.278]^*^
151–200	43 (56.6)	33 (43.4)	2.654 [1.519-4.639] ^*^	2.203 [1.130-4.296]^*^
201–250	45 (56.3)	35 (43.8)	2.619 [1.513-4.534] ^*^	1.933 [0.971-3.848]
> 250	32 (57.1)	24 (42.9)	2.716 [1.459-5.056] ^*^	2.518 [1.215-5.221]^*^
Not disclosed	45 (30.4)	103 (69.6)	0.890 [0.552-1.436]	0.748 [0.431-1.299]

^*^ *p* value < 0.05

## Discussion

Nearly half of the total households in this study stored medicines which was much lower compared to studies in countries like Indonesia, Iran, Iraq, Oman, Greece and USA where 82 to 100% of households did so [9–12, 18, 33]. This could be attributed to the difference in the economic development level between Ethiopia and the compared countries. This in turn could be translated in to difference in access to medicines, health insurance schemes as well as difference in the trend of self medication practice. However, the finding from the current study was higher compared to studies from northern Uganda as well as Addis Ababa and Tigray in Ethiopia; and was comparable to another study in southwestern Ethiopia [8, 27–29].

In terms of likelihood of storing medicines, households with members having chronic illnesses(es) were more likely to keep medicines compared to those without such individuals. Higher monthly income was also associated to higher likelihood of medicine storage. Similar findings have been reported by other studies also [8, 25].

The mean number of medicines found per household, in the current survey, was much lower compared to findings from Asia and Europe, where medicines ranging from five to 31 were found stored at home [9, 11, 12, 17, 33]. The difference here could similarly be explained by the above mentioned differences between Ethiopia and the compared countries. Studies from two towns in Ethiopia, however, reported similar number of medicines per household to the present finding [28, 29].

Households where member with chronic illness(s) lived had higher mean number of medicines compared to those with no members with such conditions, in a statistically significant manner. The nature of the diseases which require a number of medicines over an extended period of time could be a reason for this. Other variables like, presence of health professionals, highest education level in the household, monthly income of the households did not differ in a statistically significant manner with the mean number of medicines stored.

The ATC Level One categories of medicines were constituted by anti-infectives for systemic use, medicines used for the alimentary tract and metabolism as well as those used for the cardiovascular system as the top three. Studies in Uganda, Iraq, Oman and Greece reported similar findings [8, 11, 12, 33]. Findings consistent with the present study were also found by other studies in Ethiopia [27–29]. Diclofenac, paracetamol and amoxicillin ranked top among medicines found, with similar findings reported in other studies [8, 10, 28]. Most of the medicines were of solid dosage forms (80%) as also reported in other studies [8, 29].

Nearly two-thirds of the medicines kept at home were being used/taken. This was higher compared to findings in Uganda (48%), Indonesia and Iraq (31% each), [8, 9, 11]. This showed a good practice in relation to household medicines use in the town as it helps reduce wastage of useful medicines and risks from unused medicines.

Chest of drawers found in living rooms as well as bedrooms were reported as principal storages in three-quarters of the households. However, medicine cabinets dedicated for medicine storage were not found. Similarly drawers were the major storage spots as reported by another study in Ethiopia [21]. However in Iran and Oman refrigerators were reported as major storage places while a study in New Zealand reported kitchens as major storage rooms [10, 12, 16].

Almost all of the medicines assessed were advised to be acquired by physicians (83.5%) through prescriptions which was similar to a finding in Oman [12]. This could indicate a lower level of self-medication practice among the households. The finding was very much higher compared to a study in Iraq which reported only about a third of the medicines were prescribed by physicians [11].

Virtually all of the medicines found stored in the surveyed households were acquired from pharmacies be they of public, private or aid organizations ownership. This is an encouraging practice which can help minimize the risk to patients due to buying medicines of questionable quality from informal/illegal sources. Similar practices were reported by other studies [8, 9, 12].

Of medicines with recorded expiry date, 3.14% were found to be expired which was comparable to a finding by another study in Tigray region of Ethiopia [29]. However, much higher proportions of expired medicines were recorded by studies in different countries in the Middle East [11, 12, 24, 25]. The very low proportion of expired medicines could be associated to the fact that the median duration of storage of medicines was only 15 days which shows medicines were not kept for very long time in most of the households.

### Limitations

The present study did not include the rural parts of Gondar Town. The findings are not representative of the pattern of household medicine storage practice in those areas.

## Conclusions

Nearly half of the households stored medicines mostly acquired through physician advise almost entirely from dispensaries. Most of medicines kept at home were on use and were kept mostly in chests of drawers with no medicine cabinets in use. A very low proportion of the medicines were found to be expired.

The good practices should be encouraged through continued health education at health institutions and medicine retail outlets. Installing cabinets dedicated for medicine storage at households should be advocated by the town’s health bureau to improve storage conditions of medicines.

## Additional file

Additional file 1: Data collection instrument used in the data collection. (PDF 67 kb)

## Abbreviations

ANOVA: Analysis of variance
AOR: Adjusted odds ratio
ATC: Anatomical Therapeutic Chemical
CSA: Central Statistical Agency
OR: Odds ratio
SD: Standard deviation
SPSS: Statistical Packages for Social Sciences
USD: United States Dollar
WHO: World Health Organization
