# Supplementary material for: A household survey of medicine storage practices in Gondar town, northwestern Ethiopia
Source: BMC Public Health. 2017 Mar 9;17:238. doi: 10.1186/s12889-017-4152-8 (PMC5343301; doi:10.1186/s12889-017-4152-8)
Supplement: Additional file 1: — Data collection instrument used in the data collection. (PDF 67 kb) [file 12889_2017_4152_MOESM1_ESM.pdf]

University of Gondar,

College of Medicine and Health Sciences, School of Pharmacy

A data collection instrument for household survey of medicine storage practices in Gondar Town, northwestern Ethiopia

Consent Form

Dear sir/madam my name is ______________________________________. I am from the School of Pharmacy at University of Gondar working with a research team to assess the household medication storage and utilization in Gondar Town.

This interview and observation is part of the research and the information you provide will be a very important input in the understanding of the topic at hand and will help implement various activities which will base on the finding to correct problems, if any, regarding medication use and storage practices in the town.

The interview and observation will only take 10 to 15 minutes and you have a right to not answer any question that you don't want to respond to. You can also stop the interview if you want to do so. All the information from this interview will be kept confidential and used only for the study. The data will be kept using codes and analyzed anonymously in aggregate, not individually. If you have any questions about this study you can ask at any point in the interview or can call Fitsum Sebsibe Teni at 0911333607.

Are you willing to participate in this study? Yes ☐ No ☐

Respondent's code _______ Date ______________ Data collector's no. _____________

1. Socio-demographic profile and health

1.1. Kebele/sub-city_____________ 1.2. Age_____ 1.3. Sex: male☐ female ☐

1.4. Religion:

Orthodox Christianity ☐ Islam ☐ Protestantism☐ Other, specify________

1.5. Level of education:

Not able to read and write ☐ Able to read and write ☐ Primary school ☐

Secondary school ☐ College/university ☐

1.6. Ethnicity: Amhara ☐ Tigre ☐ Other, specify ___________

1.7. Occupation ___________________________

1.8. Educational level of family members: What is the highest level of education achieved by a member of the family currently living with you?

Not able to read and write ☐ Able to read and write ☐ Primary school ☐

Secondary school ☐ College/university ☐

1.9. How much is your estimate of the total family income per month (in ETB)? _____________________________

1.10. Is there anyone in the family in the health related profession? Yes ☐ No ☐

1.11. Is there anyone in the family suffering from long term/chronic conditions? (e.g. hypertension, diabetes)

Yes ☐ No ☐

If yes, please specify the condition ____

2. Current medicine use and storage

2.1. Do you currently keep medicines at home?

Yes ☐ No ☐

2.2. If yes, are you willing to show it/them to me?

Yes ☐ No ☐

Medicine number	2.3. Medication name, strength, dosage form	2.4. Current status of medicine "√"				2.5. Purpose of use (illness)
		On use		Kept for future use	Not for use	
		Original /first user	Another person			

Medicine number	2.6. Who advised to get the medicine?	2.7. How was it acquired? "√"		2.8. From where? (Source)	2.9. When/How long has it been?	2.10. Why was this source chosen?
		With Prescription	Without prescription			

Medicine number	2.11. Type of package		2.12. Packaging material *	2.13. Expiry date "√"			2.14. Storage place
	Original	Other		Recorded		Not recorded	
				Expired	Not expired		

*Type of package: A: Plastic bag , B: Paper bag C: Blister pack D: Strip pack E: Bottle F: Vial G: Other (specify)

Thank you!
